# Resection of isolated brain metastases in non-small cell lung cancer (NSCLC) patients – evaluation of outcome and prognostic factors: A retrospective multicenter study

**DOI:** 10.1371/journal.pone.0253601

**Published:** 2021-06-28

**Authors:** Julia Fuchs, Martin Früh, Alexandros Papachristofilou, Lukas Bubendorf, Pirmin Häuptle, Lorenz Jost, Alfred Zippelius, Sacha I. Rothschild

**Affiliations:** 1 Medical Oncology, Department Internal Medicine, University Hospital Basel, Basel, Switzerland; 2 Department of Medical Oncology and Hematology, Cantonal Hospital St. Gallen, St. Gallen, Switzerland; 3 Department of Medical Oncology, University Hospital Bern, Bern, Switzerland; 4 Lung Cancer Center Basel, Comprehensive Cancer Center, University Hospital Basel, Basel, Switzerland; 5 Department of Radiation Oncology, University Hospital Basel, Basel, Switzerland; 6 Institute of Pathology, University Hospital Basel, Basel, Switzerland; 7 Department Oncology, Hematology and Transfusion Medicine, Cantonal Hospital Baselland, Liestal, Switzerland; 8 Medical Oncology, Cantonal Hospital Baselland, Bruderholz, Basel, Switzerland; Baylor College of Medicine, UNITED STATES

## Abstract

**Background and objectives:**

Brain metastases occur in about 30% of all patients with non-small cell lung cancer (NSCLC). In selected patients, long-term survival can be achieved by resection of brain metastases. In this retrospective study, we investigate the prognosis of NSCLC patients with resected brain metastases and possible prognostic factors.

**Methods:**

In 119 patients with NSCLC and resected brain metastases, we report the following parameters: extent of resection, resection status, postoperative complications and overall survival (OS). We used the log-rank test to compare unadjusted survival probabilities and multivariable Cox regression to investigate potential prognostic factors with respect to OS.

**Results:**

A total of 146 brain metastases were resected in 119 patients. The median survival was 18.0 months. Postoperative cerebral radiotherapy was performed in 86% of patients. Patients with postoperative radiotherapy had significantly longer survival (median OS 20.2 vs. 9.0 months, p = 0.002). The presence of multiple brain metastases was a negative prognostic factor (median OS 13.5 vs. 19.5 months, p = 0.006). Survival of patients with extracerebral metastases of NSCLC was significantly shorter than in patients who had exclusively brain metastases (median OS 14.0 vs. 23.1 months, p = 0.005). Both of the latter factors were independent prognostic factors for worse outcome in multivariate analysis.

**Conclusions:**

Based on these data, resection of solitary brain metastases in patients with NSCLC and controlled extracerebral tumor disease is safe and leads to an overall favorable outcome. Postoperative radiotherapy is recommended to improve prognosis.

## Introduction

Lung cancer remains the leading cause of cancer death worldwide [1]. Brain metastases occur in 20% to 32% of patients diagnosed with NSCLC [2, 3], most frequently in patients with adenocarcinomas and tumors harboring EGFR mutations or ALK rearrangements with an incidence of up to 72% [4–6]. Brain metastases are associated with significant morbidity and poor survival outcomes [7]. On the other hand, in some patients, brain metastases may not be clinically significant and remain undetected as shown in autopsy series where the incidence is reported to be up to 60% in unselected NSCLC patients [8]. Overall, the prognosis of patients with brain metastases has improved during the last two decades, likely due to better prognosis and improved and more frequent radiological imaging. Based on a recent report of the GPA for lung cancer using molecular markers (Lung-molGPA) median survival of patients with brain metastases diagnosed between 2006 and 2014 ranges from 3 to 46.8 months [9]. The wide range of survival reflects the overall better outcome of patients with oncogenic alterations, mainly EGFR mutations and ALK translocations.

Treatment of NSCLC patients with brain metastases is still controversial. As the presence of brain metastases is often an exclusion criterion in clinical trials most of the evidence is retrospective in nature. Moreover, most of the literature discusses symptomatic brain metastases, as routine baseline brain imaging is often not done in asymptomatic patients outside of clinical trials [10]. It is common practice to treat solitary or single brain metastases in patients with good performance status and controlled extracranial disease with surgery and postoperative radiation therapy, mainly stereotactic radiotherapy to the resection cavity [11] or with stereotactic ablative radiotherapy (“radiosurgery”) [12]. Long-term survival after surgical treatment of metachronous brain metastases in patients with primary NSCLC has been reported in several studies [13, 14]. Mussi et al. describe a higher survival rate in patients with metachronous brain metastases compared to those with synchronous metastases. However, also in this regard there are controversial data [15]. For patients with metachronous brain metastases the length of the median interval between the two surgical procedures (≥14.5 months) seems to be an important prognostic factor [14]. The results of surgical treatment of synchronous brain metastases along with the primary tumors is even more controversial [15, 16]. Our aim was therefore to assess the prognosis in an unselected population of NSCLC patients with brain metastases that had been resected and to describe possible clinical characteristics associated with the outcome.

## Patients and methods

### Patients

All patients with a histo-pathological diagnosis of NSCLC undergoing surgical resection of brain metastases between 2000 and 2014 at two centers in Switzerland were included in this retrospective analysis. This study was performed in accordance with institutional review board and was approved by the local ethical committee (Ethical Committee Northwestern and Central Switzerland, EKNZ). Due to the retrospective analysis no patient consent was necessary. All data were recorded in coded manner according to the requirements of the ethics committee.

### Data acquisition

Patient- and tumor-specific data were obtained from the patients’ medical records. Stage was assessed according to TNM 6th edition. Overall survival (OS) was measured from the time of diagnosis (stage IIIB, IV or inoperable situation) to date of death, or date of last patient contact if lost to follow-up. Furthermore, we also assessed OS from the timepoint of occurrence of brain metastases.

### Statistical analysis

Qualitative variables were summarized by count and percentage, quantitative variables by mean, median, and range. Fisher exact and Chi-square tests were used to assess correlations of nominal covariate distributions and response groups. Probabilities of survival were calculated using the Kaplan-Meier estimator. Survival curves were compared using the log-rank test in univariate analysis. Multivariable Cox regression was used to investigate potential prognostic factors. A two-sided p<0.05 was considered statistically significant.

## Results

One hundred and nineteen consecutive patients with brain metastases from NSCLC undergoing surgical resection between 2000 and 2014 were analyzed.

### Patients

The median age at diagnosis was 59.5 years (rage: 34.5–89.5 years), 56% (n = 67) of the patients were male and 44% (n = 52) were female. Seventy-four percent of patients were current or former smokers. The majority of patients had adenocarcinoma histology (55%, n = 65). Information on EGFR mutation status was available in 33 patients with adenocarcinoma. Only two patients had an EGFR mutation, one patient (female, 57 years) had an EGFR exon 20 insertion (p.N771DelinsGY) and one patient (female, 77 years) had an activating EGFR mutation (del19). Eighty-one patients (68%) were diagnosed with brain metastases at the first occurrence of NSCLC (synchronous metastases). For this analysis, we defined synchronous metastases as metastases detected within 60 days of lung cancer diagnosis. Overall, mean time to the occurrence of brain metastases was 171 days (range, 0–1535 days). In patients with metachronous metastases the mean time to the occurrence of brain metastases was 528 days (range: 134–1535 days). In 77 patients (65%) the initial site of metastatic disease was the brain. At the time of diagnosis of brain metastases, 38 patients (32%) had also extracranial metastases. Twenty-seven of these patients (71%) with extracranial metastases had only 1 additional metastatic side. Eleven patients (29%) had more than one additional metastatic side, with median number of 3.6 (range: 3–6). Forty-seven patients (40%) had a surgical resection of the primary tumor. Seventy patients (59%) had additional systemic therapy and these patients received a median of 1.9 lines (range 1–7 lines). Of these patients 42 patients (60%) received one line of systemic therapy, ten patients (14.3%) two and three lines, respectively and eight patients (11.4%) more three lines. Baseline characteristics of the patients are summarized in Table 1.

**Table 1:** Patient characteristics.

Characteristic	Median (range) or number of patients (%)
Age at diagnosis, years	59.5 years (34.5-89.5)
Gender	
• Male	67 (56%)
• Female	52 (44%)
Histological subtype	
• Adenocarcinoma	65 (55%)
• Squamous cell carcinoma	21 (18%)
• Large cell carcinoma	6 (5%)
• NOS	16 (13%)
• Other	11 (9%)
Smoking History	
• Current	39 (33%)
• Former	49 (41%)
• Never	7 (6%)
• Unknown	24 (20%)
Initial stage	
• I	7 (6%)
• II	10 (8%)
• IIIA	8 (6%)
• IIIB	5 (4%)
• IV	81 (68%)
• Unknown	9 (8%)

At the time of analysis, 98 patients (82.4%) were deceased. Among the 21 living patients, the median follow-up time was 50.2 months. Eight patients were lost to follow-up. The median OS was 18.0 months. One year survival rate was 63%. From the timepoint of detection of brain metastases median OS was 13.8 months. Twelve percent (n = 14) of patients were alive after five years. Of the 99 patients who died, 37 patients (37.4%) were proven to have died of tumor progression. Three patients (3.0%) died of non-tumor related causes, for the remaining 59 patients (59.6%) the cause of death was not documented.

### Characteristics and treatment of brain metastases

In total, 146 brain metastases were resected in 111 patients; the median number of resected metastases was 1 (range: 1–4). Ninety patients (76%) had a solitary brain metastasis and 21 patients (17%) had at least two metastases, in 7% (8 patients) the number of brain metastases was unknown. Largest median diameter of resected metastases was 25 millimeters (range: 6–70 mm). The majority of the patients had metastases localized in the frontal cortex or cerebellum (51%, n = 61). One hundred and two patients (86%) received postoperative radiotherapy. Sixty-six percent (n = 77) of patients were treated with whole brain radiotherapy (WBRT), 35% (n = 36) received stereotactic radiotherapy (SRT). 66 patients (65%) received WBRT alone, 25 patients (25%) were treated with SRT alone, 11 patients (11%) received WBRT and additional SRT. Eleven patients (9%) did not receive any postoperative radiotherapy. The most common reason for not performing postoperative radiotherapy was postoperative complications or a long recovery time after surgery. The median dose of postoperative radiotherapy was 28 Gy (range: 8–86 Gy). Characteristics and treatment patterns of brain metastases are summarized in Table 2.

**Table 2:** Characteristics of brain metastases.

Characteristic	Median (range) or number of patients (%)
Number of brain metastases	
• 1	90 (76%)
• 2	11 (9%)
• ≥ 3	10 (8%)
• Not available	8 (7%)
Largest diameter	
• ≤ 10 mm	11 (9%)
• 11–30 mm	46 (39%)
• 31–50 mm	29 (24%)
• > 50 mm	5 (4%)
• Unknown	28 (24%)
Additive Radiotherapy	
• Yes	102 (86%)
• No	11 (9%)
• Unknown	6 (5%)
Type of Radiotherapy	
• Whole brain radiotherapy	66 (65%)
• Stereotactic radiotherapy	25 (25%)
• Whole brain radiotherapy + stereotactic boost	11 (11%)
Median dose of postoperative radiotherapy	28 Gy (8–86)

### Patient outcome and prognostic factors

**Fig 1:**
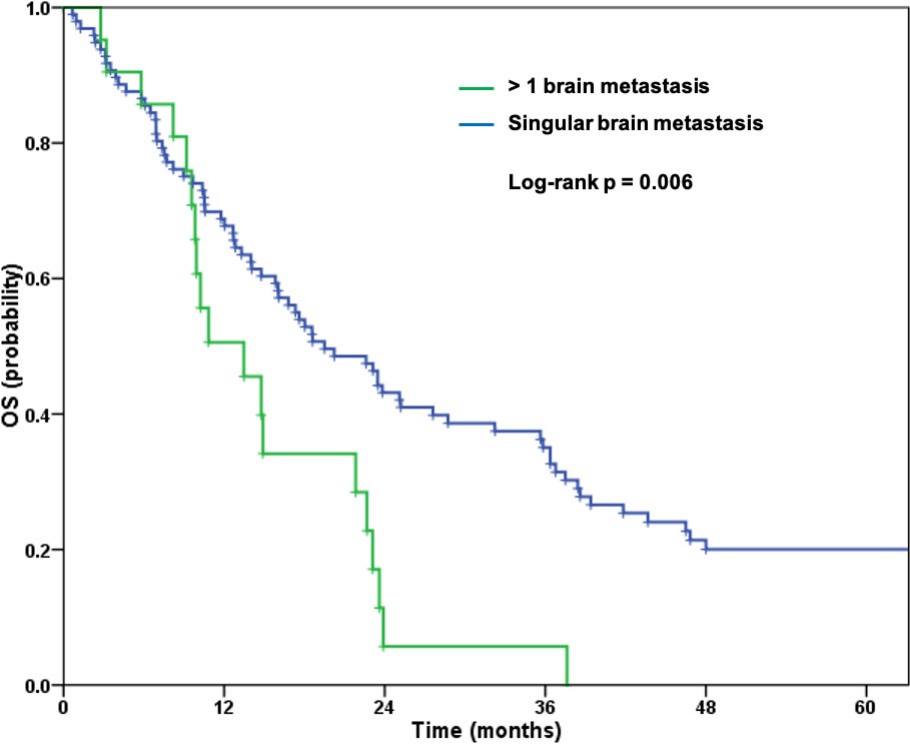
Overall survival for patients with one or more brain metastases.

**Fig 2:**
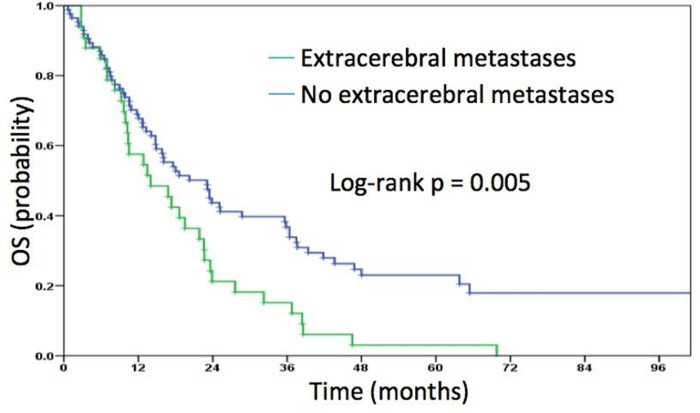
Overall survival for patients with or without extracerebral metastases.

Patients with more than one brain metastasis had a significantly worse outcome compared to those with a singular metastasis (median OS 13.5 vs. 19.5 months, p = 0.006) (Fig 1). Furthermore, patients with extracerebral metastases had a significantly poorer outcome (Fig 2). Median OS for patients with extracerebral metastases was 14.0 months compared to 23.1 months for patients with brain metastases only (p = 0.005).

**Fig 3:**
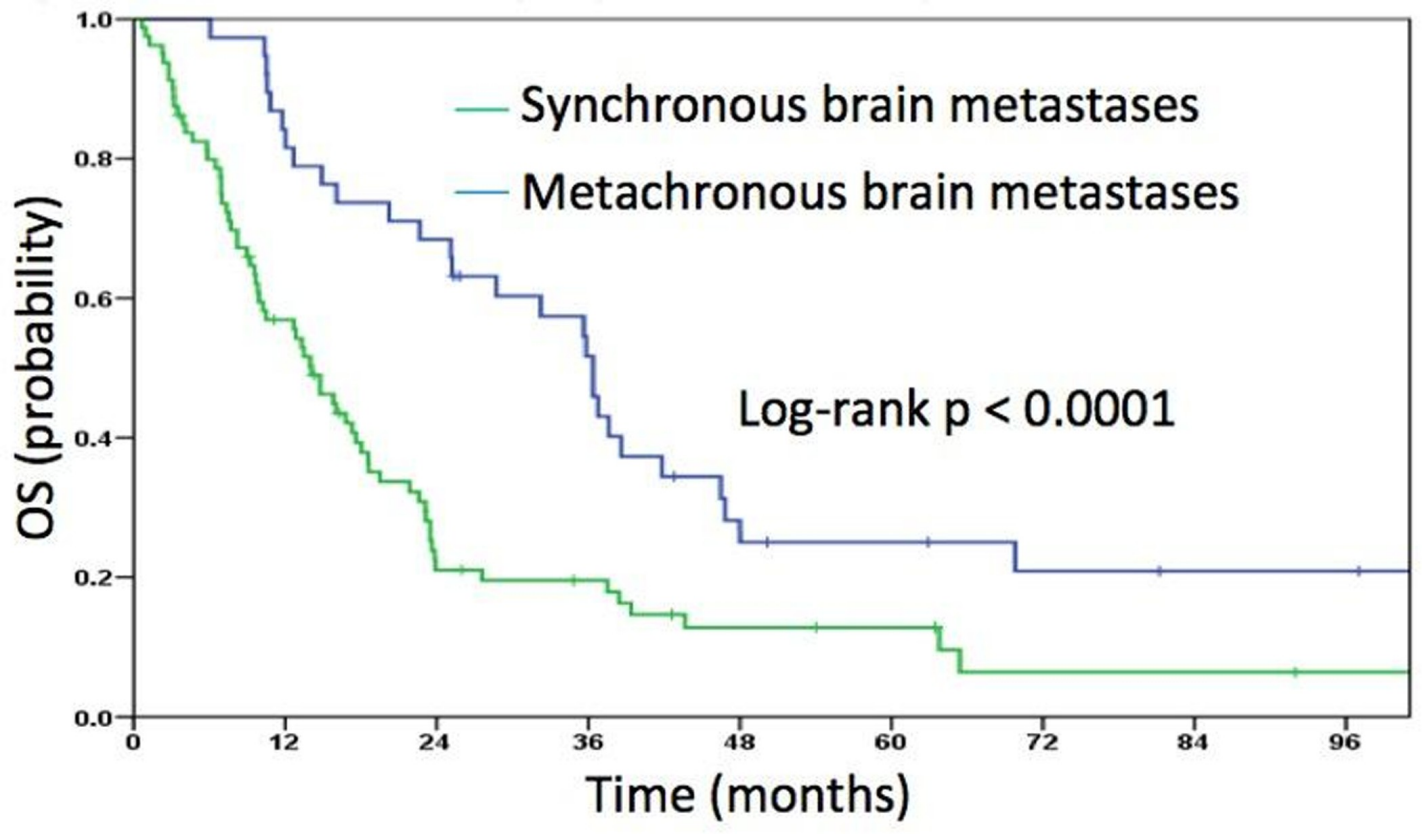
Overall survival for patients with synchronous and metachronous metastases.

Comparing the overall survival of patient with synchronous and metachronous brain metastasis performing a log rank test shows that patients with synchronous metastases had a significantly worse outcome (median OS 24.1 vs. 36.4 months, p<0.001) (Fig 3).

**Fig 4:**
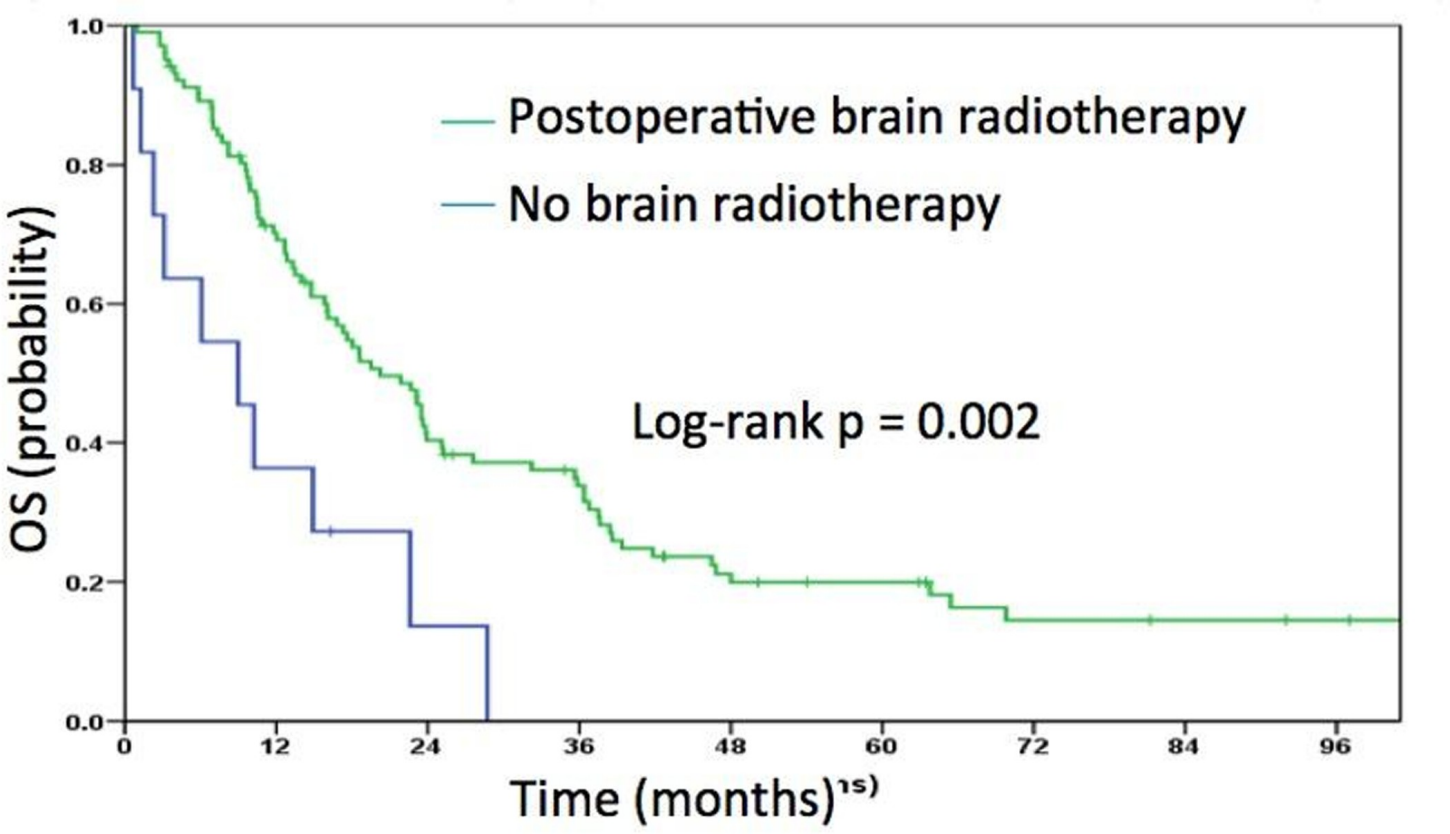
Overall survival for patients treated with or without postoperative radiotherapy.

Patients treated with postoperative radiotherapy had a median OS of 20.2 months compared to nine months in patients treated with surgery alone (p = 0.002) (Fig 4).

Eighty-seven patients (73%) suffered from non-squamous cell carcinoma. These patients had a significantly longer survival compared to patients with squamous-cell histology: median OS 22.6 vs. 12.0 months (p = 0.019). The 77-year old female patient with the activating EGFR mutation had three synchronous brain metastases and underwent WBRT with 30 Gy after resection of all metastatic lesions. She passed away 27.6 months after the initial diagnosis due to tumor progression while receiving erlotinib as first-line palliative therapy. The 57-year old female patient with the EGFR Exon 20 insertion mutation had synchronous brain metastases as well as visceral metastases. She underwent resection of two brain metastases and postoperative WBRT with 30 Gy. After several lines of palliative chemotherapy, the patient died due to septic shock 14 months after the primary diagnosis.

For the univariate analysis we included clinical and treatment-related factors. The results of univariate analysis are shown in Table 3.

**Table 3:** Univariate analysis.

Factor	Variable	Median OS (months)	p-value
Histological subtype	Squamous cell carcinoma	12.0	0.019
	Non-squamous cell carcinoma	22.6	
Extracerebral metastases	yes	14.0	0.005
	no	23.1	
> 1 brain metastasis	yes	13.5	0.006
	no	19.5	
Additive radiotherapy	yes	20.2	0.002
	no	9.0	

We tested significant variables from the univariate analysis for independence in a multivariate analysis. In the multivariate analysis the presence of extracerebral metastases and resection of more than one brain metastasis were significant independent prognostic factors for poorer outcome (Table 4). Squamous cell histology was of borderline significance whereas postoperative radiotherapy did not predict outcome in the multivariate analysis.

**Table 4:** Multivariate analysis.

Factor	Hazard Ratio	95% CI	p-value
>1 brain metastasis	1.7	1.0–3.0	0.05
Additive radiotherapy	1.1	0.9–1.2	0.12
Extracerebral metastasis	1.6	1.1–2.6	0.034
Squamous histology	1.6	0.9–2.6	0.082

### Subgroup of patients with an overall survival of more than 5 years

In terms of an exploratory analysis, we analyzed those patients who had an overall survival of more than five years. In these 14 patients, the median age at the time of diagnosis was 56.9 years (range: 40–89 years). Ten of these patients (71%) were female. The majority of these patients had adenocarcinoma (n = 11, 79%). Mean time to occurrence of brain metastases was 309 days (range: 0–1155 days), six patients (43%) had synchronous cerebral metastases. None of these patients had additional extracerebral metastases. All of these patients had a single brain metastasis, the mean diameter was 34.2 mm (range: 10-70mm). Twelve patients (86%) received additional radiotherapy, thereof ten patients WBRT and two patients WBRT and SRT.

### Postoperative complications

Twenty-four percent (n = 29) of patients experienced postoperative complications, including need for surgical reintervention (6%), neurological deficits (4%), infection (4%), stroke (3%) and others (12%). The 30-day postoperative mortality rate was 6% (seven patients) The occurrence of postoperative complications was not associated with the overall outcome.

## Discussion

The incidence of brain metastases is proportionally higher in lung cancer than in other solid tumors [3] with significant impact on patients performance status and prognosis [17]. Brain metastases in NSCLC patients is therefore a common clinical problem. There is controversy regarding the treatment of brain metastases. Even though the surgical removal of individual metastases in patients with extracerebral controlled disease is an accepted standard of care, there are numerous unanswered questions, especially with regard to patient selection. With our retrospective analysis of patients, we are able to show that the resection of brain metastases with subsequent radiotherapy is a valid therapeutic option that may lead to long-term survival in some patients.

Surgical resection has become an accepted treatment option for patients with single brain metastases after the publications of several studies evaluating the role of brain metastases resection [18–21]. Three of four randomized studies demonstrated that surgical resection in addition to WBRT resulted in prolonged survival among patients with a single brain metastasis. In the study by Patchell et al. [18] patients undergoing surgery had also a better quality of life. Conversely, high rates of local recurrence (50%-60% at 12 months) have been observed in patients treated with surgery alone without postoperative radiotherapy [22–24]. These studies demonstrated improved local control but no significant difference in survival. The European Organization for Research and Treatment of Cancer (EORTC) 22952–26001 study is the largest prospective trial of SRS or metastasectomy with or without WBRT in the setting of limited brain metastases, finding no impact of WBRT on survival [22]. In a later analysis for patients with limited extracerebral disease and one to three cerebral metastases, no benefit of additional WBRT was seen either, and the authors concluded that WBRT should be omitted in this situation [25]. Due to the neurocognitive toxicity of WBRT, the interest in SRT to resection cavities with either single fraction [26–28] or multiple fractions [29] has recently increased. A recent randomized study of SRT vs. WBRT in patients with a resected brain metastasis with a diameter of the postoperative cavity of less than 5 cm showed no difference in survival but less cognitive impairment in patients treated with SRT [30]. However, the rate of intracranial tumor control was lower for SRT, a finding largely due to an increased incidence of the occurrence of new brain metastases in patients without WBRT. In another recently published study, the efficacy of postoperative stereotactic irradiation was confirmed [31]. Controlled primary tumor and the presence of a singular brain metastasis were independent predictors for this therapeutic approach. In our study the majority of patients received postoperative WBRT. We could find a significant overall survival benefit for patients treated with postoperative radiotherapy. However, in the multivariate analysis postoperative radiotherapy was not an independent prognostic factor for treatment outcome. We hypothesize, that in our study patients with a more favorable prognosis were selected for postoperative radiotherapy introducing a potential selection bias.

Several studies compared surgical treatment of metachronous and synchronous brain metastases regarding overall survival. Some showed a worse outcome for patients suffering from a synchronous brain metastasis [14, 32, 33] comparable to the findings in our study. However, other studies could not confirm the prognostic role of the time point of occurrence of brain metastases [13, 15, 34, 35]. Interestingly, in our study, 6 out of 14 patients surviving more than five years had synchronous brain metastases.

Concerning the survival of patients with single brain metastasis compared to those with multiple metastatic lesions Wroński et al. [33] documented a significant difference between the two groups with a longer overall survival for patients with single brain metastasis (11.1 vs. 8.5 months, p<0.02). These results are in line with our findings, showing a significant longer survival for patients with singular cerebral metastases. Patients with a controlled primary tumor without extracerebral metastases have a better prognosis and may benefit the most from radical local therapy [8].

Addressing prognostic factors and the histologic type of cancer, some studies show a better prognosis of patients with adenocarcinoma, compared to those with squamous cell carcinoma [36, 37]. While Burt et al. [37] suggested an improved survival of patients with adenocarcinoma, these findings were not statistically significant. Bonnette et al. [36] however, was able to show a significantly prolonged survival time in patients with adenocarcinoma compared to those with squamous cell carcinoma or large cell carcinoma (p = 0.019). In contrast, Mussi and associates [14] found that the presence of squamous lung cancer was the only variable that was significantly associated with a longer survival (p = 0.02). In our study patients with non-squamous cell carcinoma had an improved overall survival compared to those with squamous-cell histology, which is in agreement with the findings of Bonnette et al. Yet, histology was not an independent negative prognostic factor in a multivariate analysis in our study. Importantly, only two patients had driver alterations in our analysis. Therefore, based on our data, we cannot draw conclusions about the role of metastasectomy in patients with therapeutically amenable molecular driver alterations.

The overall complication rate in patients undergoing surgical resection of brain metastases range from 5% up to 40% [18, 21, 32, 38–40]. In our retrospective analysis 21% of patients had postoperative complications. The postoperative mortality rate in our cohort was 6%. Although the postoperative complication rate in our cohort is comparable to published data, this fact should be included in decision making in the management of NSCLC patients with brain metastases.

Improved radiooncological techniques, in particular the possibility of stereotactic irradiation of brain metastases, has raised the question whether stereotactic irradiation alone leads to an equally good oncological outcome instead of surgery with the corresponding neurological risks. An exploratory analysis of the multi-institutional European Organisation for Research and Treatment of Cancer (EORTC) trial found that local recurrence rates were similar between stereotactic radiotherapy and surgery [12]. However, when stratified by interval, patients after surgery had a much higher risk of early local recurrence (0-3months) compared with those undergoing stereotactic radiotherapy, although specifically, the likelihood of local recurrence was lower after 9 months in the surgery group. Most studies comparing surgery and SRS report similar outcomes; however, they are not randomized and are likely to be affected by selection bias [41–43]. Careful and interdisciplinary individual evaluation is needed to determine the optimal therapeutic procedure in patients with cerebral metastases of NSCLC. The current guidelines of the European Association of Neuro-Oncology (EANO) recommend stereotactic radiosurgery for metastases less than 3–3.5 cm in diameter and surgical resection for a limited number of metastases (1 to 3) if the extracerebral disease is controlled and the patient’s general condition permits [44].

### Limitations

Our study is a retrospective analysis, which creates the risk of selection bias. We cannot rule out, or have to assume that the decision/indication for resection of brain metastases and mainly also for postoperative radiotherapy was due to performance status and other prognostic factors (e.g. age). Nevertheless, our analysis confirms the role of surgery in NSCLC patients with singular brain metastases as this might lead to long-lasting disease control and an overall survival of more than five years in a subset of patients.

Furthermore, another bias of all retrospective analysis is the incompleteness of medical records. However, we have comprehensive data on overall outcome with only very few patients (n = 8) lost to follow-up. Another factor decreasing the risk of selection bias in our cohort is the fact that we included all consecutive NSCLC patients with resected brain metastases without any further selection. In addition, a limitation of our work is the lack of data on the accepted risk scores (e.g., RPA, GPA) and the lack of data on local tumor control after resection.

The percentage of patients who received postoperative stereotactic radiation alone in our analysis is 25%. Due to the fact that 57 patients (51%) had a metastasis of less than 30 milimeter size, a higher rate of stereotactic irradiation would be expected nowadays. In these patients nowadays, a critical evaluation of the place value of metastasectomy would also have to be discussed.

We report on a cohort of NSCLC patients in the pre-immunotherapy era. immune-checkpoint inhibitors (ICI) are being rapidly adopted into clinical practice for patients with metastatic NSCLC and have shown intracranial activity similar to the response rates outside the brain [45, 46]. Therefore, our data can serve as a basis for future analyses considering the intracerebral activity of ICI.

## Conclusions

In conclusion, our study demonstrates the significance and safety of a surgical intervention in NSCLC patients with brain metastases without molecular driver alterations. We were able to show that with an interdisciplinary therapeutic approach, some of these patients can even achieve long-term survival. After the resection of cerebral metastases, patients should be offered consolidative radiotherapy. Prospective trials are needed to characterize the patient population experiencing the greatest benefit from a surgical procedure. This issue needs to be further investigated in the light of new treatment options such as ICI and newer targeted therapies for patients with oncogenic driver mutations.**Acknowledgments**
